# Changes of gait pattern in children with Charcot-Marie-Tooth disease type 1A: a 18 months follow-up study

**DOI:** 10.1186/1743-0003-10-65

**Published:** 2013-07-02

**Authors:** Maurizio Ferrarin, Tiziana Lencioni, Marco Rabuffetti, Isabella Moroni, Emanuela Pagliano, Davide Pareyson

**Affiliations:** 1 Biomedical Technology Department, Don Carlo Gnocchi Foundation Onlus IRCCS, Milan, Italy; 2 Division of Child Neurology, IRCCS Foundation, Carlo Besta Neurological Institute, Milan, Italy; 3 Developmental Neurology Division, IRCCS Foundation, C. Besta Neurological Institute, Milan, Italy; 4 Unit of Clinic of Central and Peripheral Degenerative Neuropathies, IRCCS Foundation, Carlo Besta Neurological Institute, Milan, Italy

**Keywords:** Gait analysis, Charcot-Marie-Tooth disease, Toe walking, Heel walking, Foot-drop, Push-off, Children, Follow-up

## Abstract

**Background:**

In a previous study we identified 3 different gait patterns in a group of children with CMT1A disease: Normal-like (NL), Foot-drop (FD), Foot-drop and Push-off Deficit (FD&POD). Goal of the present study was to perform a follow-up evaluation of the same group of patients to analyze possible changes of gait features in relation to disease progression or specific therapy.

**Methods:**

Nineteen children with CMT1A were evaluated clinically (CMT-Examination Score and Overall Neuropathy Limitation Scale) and through gait analysis 18.2±1.5 months after a baseline evaluation. Meanwhile, 3 of them had foot surgery.

**Results:**

Fifteen out of the 16 non-operated patients significantly changed at least one of the two parameters associated to primary signs (FD and/or POD). Eleven participants worsened at least one parameter and 9 improved one parameter. CMTES significantly worsened for the group of non-operated patients. However, there was no change in CMTES score in 4 patients and in ONLS score in 11. At subgroup level, participants originally belonging to NL group showed a trend towards a foot-drop deficit (−15%, ns); FD and FD&POD subgroups did not change their primary signs, although significant changes were identified individually. All 3 patients operated have improved push-off and proximal joint patterns during walking. Clinical scores did not change within any sub-group.

**Conclusions:**

Subtle changes occurring in 1.5 year in gait features of CMT1A children can be instrumentally identified. Such changes show a large inter-subject variability, with some patients even improving their walking pattern. There is anecdotal evidence that foot surgery may improve the push-off phase of gait.

## Background

Charcot-Marie-Tooth type 1A (CMT1A), an inherited demyelinating neuropathy, is the most common hereditary neuropathy (40-50% of all CMT cases). It is characterized by length-dependent degeneration of the motor and sensory fibres with consequent weakness of distal limb muscles, distal sensory loss and foot deformities [1]. Usually, symptoms start in childhood and then slowly progress centripetally, from the intrinsic foot muscles to the leg muscles, thus affecting locomotor functions. Several clinical and neurofunctional measures have been proposed [2]–[5] to monitor the progression of impairment, disability and quality of life in CMT patients. However, standardized measures for routinary clinical evaluation of the pediatric CMT population are lacking [3,6] and only recently a pediatric scale was proposed, validated and its responsiveness is in progress [7].

Gait analysis has been used to objectively classify walking patterns in adults [8,9] and children [10] with CMT disease. These studies identified CMT-related typical gait abnormalities, i.e. foot-drop and push-off deficit, and consequent locomotor strategies to compensate for such distal signs. Gait analysis was also used to evaluate the functional efficacy of different orthoses for adults with CMT [11], finding that specific orthotic management may control effectively foot-drop and increase gait speed, besides improving compliance with respect to standard Ankle Foot Orthoses.

Longitudinal studies of CMT disease are important to analyze the progression of the disease, to improve the accuracy of prognosis and to better assess the efficacy of new therapy in intervention studies [1]. A few prospective natural history studies are reported in literature describing a slow progression of impairment and disability in CMT1A [2,12,13].

However, to our knowledge, no longitudinal study on CMT patients using gait analysis has been published yet, although it was shown to be a useful tool in longitudinal studies both on normal children during growing [14] and on pathological cases, for instance patients after total knee replacement [15] or children with Cerebral Palsy to evaluate the natural progression of gait [16].

In a previous study [10] on a group of 21 children with CMT1A we identified 3 different gait patterns, through a cluster analysis technique on gait analysis parameters related to primary signs (foot-drop and push-off deficit). Accordingly, participants were classified as 1) normal-like (NL), when no primary gait deviations were detected; 2) patients with foot-drop (FD), when the only significant alteration with respect to controls was the deficit of ankle dorsiflexion during swing; 3) patients with foot-drop and push-off deficit (FD&POD), when a significant reduction of plantarflexion power at push-off was evidenced in addition to foot-drop. Patients belonging to the NL subgroup showed ankle dorsiflexion deficit during heel-walking. The FD&POD subgroup was associated to a significantly worse clinical score.

Aim of this study was to perform a 1.5 year follow-up assessment on the same group of CMT1A young patients to verify whether changes in locomotor functions due to disease progression or to specific therapies were detected by gait analysis techniques and to describe their possible correlation with changes in clinical scores. Moreover, we aimed to test whether the progression of this degenerative condition was associated to the shift of patients towards more severe gait pattern classification.

## Methods

### Participants

The group analyzed in the present study consisted of nineteen children with CMT1A (9 females, 10 males; mean±SD: age 11.8±2.8 years; body mass 43.0±12.1 kg; height 151±15 cm; CMTES ^a^ 4.0±2.5; ONLS ^b^ 2.0±1.0; time from first clinical symptoms: 7.3±3.8 years) all belonging to the group considered in the baseline study [10]. Inclusion criteria for the baseline study were age < 18 yrs, diagnosis of CMT1A based on clinical and genetic criteria. Exclusion criteria were presence of other neurological diseases or unrelated clinical conditions affecting locomotor functions; inability to walk unaided barefoot; previous double or triple arthrodesis of the rear foot joints.

Of the original twenty-one children included in the baseline study, two (one originally belonging to the NL subgroup and one to the FD subgroup) could not be reassessed, three (all originally belonging to the FD&POD subgroup) underwent foot surgery between the baseline and the follow-up evaluation and will be discussed separately, the other sixteen allowed for the study of the disease natural progression. The average time between baseline and follow-up evaluations was 18.2±1.5 months.

Data from eighteen healthy age-matched children (9 females, 9 males; mean±SD: age 11.0±3.3 years; body mass 41.4±14.3 kg; height 146±22 cm) were used as control reference. All participants gave informed written consent and the protocol was approved by the local Ethics Committee.

### Instrumentation, protocol and data analysis

Kinematic data were collected using a 9-camera SMART-D motion capture system (BTS, Milano, Italy) sampling at 200 Hz; two consecutive force plates (Kistler, Winterthur, Switzerland), with 800 Hz sampling frequency, provided ground reaction forces.

The total-body LAMB marker set [17] was adopted, which included 29 retroreflective markers (12 mm diameter) positioned on the head, upper limbs, trunk, pelvis and lower limbs.

CMT1A patients were asked to perform 5 trials at their natural speed (task NW); controls performed 15 trials at different speeds. Control trials whose speed was inside the speed range observed in patients were selected as normative reference. Additional tasks included toe-walking and heel-walking, performed at self-selected speed and with an effort to maximize the lift of the heel or toe from the ground during walking.

Data elaboration are fully described in [10] and included: low pass filtering of markers’ coordinates, computation of internal joint centers, calculation of lower limb joint kinematics and kinetics in all anatomical planes. Specific values were selected for each variable, according to their significance and relation to specific clinical signs. For each patient and side, the average value of selected parameters and the average pattern of kinematic/kinetic variables across five trials were computed. Biomechanical data from right and left sides were not averaged; instead, according to Burns et al. [18], we focused data analysis on the dominant side or, in case of asymmetry of musculoskeletal lower limb involvement, on the most affected side at the baseline evaluation.

The described experimental procedure and data analysis was adopted for both baseline and follow-up evaluation. Length and mass related parameters were normalized to body height and weight respectively, as suggested by several studies [19]–[21] to remove any effect due to these two age-dependent factors and thus allowing for a direct comparison between evaluations made 18 months apart on growing subjects. The follow-up evaluation was not performed in children belonging to the control group, because it was demonstrated that, in normal subjects with mature gait (like those considered in the present study), gait kinematics and kinetics are characterized by walking speed, not age [14]. This further supports the use of speed-matched control data as normative reference.

### Parameters

We focused on the same biomechanical parameters reported in the baseline study: spatio-temporal gait parameters (gait speed, stride length and cadence), range of motion (ROM) of lower limb joints and two indexes of disease-related distal deficits (AROM_ratio_ and AW_st_). AROM_ratio_, calculated as the ankle dorsiflexion ROM during swing phase divided by the ROM over the whole gait cycle, is related to foot-drop deficit and is expected to be smaller in CMT1A patients than in healthy subjects. AW_st_ is the positive ankle work (calculated as integral of power curve, normalized to body weight) in stance phase, thus accounting for the push-off mechanism. The latter two parameters, related to primary gait deviations of patients with CMT, were used to classify each patient into one of the three identified clusters (NL, FD and FD&POD). Finally, to quantify the reduced capabilities in more demanding tasks, the difference between the mean ankle angle of toe- and heel-walking (ATH_Δmean_) was computed.

All these parameters showed good reliability in CMT patients [22].

Normality of parameters distribution was tested with Shapiro-Wilk Test and was verified for biomechanical parameters but not for clinical scores, thus significance of differences between baseline and follow-up evaluation was tested with parametric tests (paired T-test) for the former and with non parametric tests (Wilcoxon Signed Rank test) for the latter. Non parametric Spearman’s Rho coefficient was computed to test for correlation between baseline-to-follow-up changes of biomechanical parameters and of clinical scores. To assess significance of mean differences between controls and the whole group of CMT1A patients T-test was applied on biomechanical parameters. Finally, comparisons among controls and CMT1A sub-groups were performed with ANOVA and Tukey’s HSD post-hoc for biomechanical parameters and with Kruskas-Wallis ANOVA and Nemenyi-Damico-Wolfe-Dunn post-hoc for clinical scores. All tests have been performed with R software ver. R2.14.1 (http://www.R-project.org) setting a significance level of 0.05, with a proper correction for multiple comparisons in post-hoc tests.

**Figure 1:**
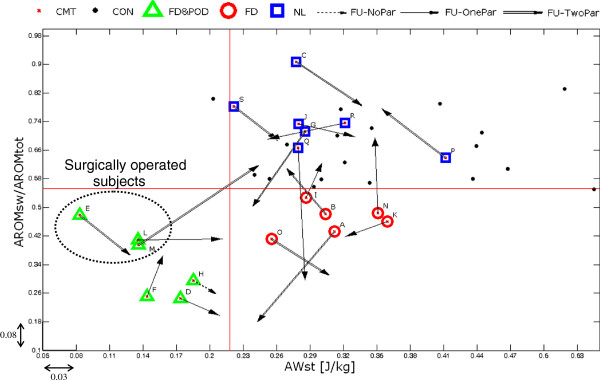
**Representation, for each CMT1A patient, of changes of parameters associated to primary signs between the baseline (empty shapes) and the follow-up (arrow tips) evaluation.** The threshold values of significance change for each parameter, 0.08 for AROM_ratio_ and 0.03 J/kg for AW_st_, are reported close to the correspondent axes. Double arrow = both parameters changed significantly, single arrow = only one parameter changed significantly, dashed arrow = no parameter changed significantly. Healthy controls are reported as filled circle. The horizontal and vertical red lines represent the 5^th^ percentile values in the control subjects’ distribution of the two primary parameters associated, respectively, to foot-drop (AROM_sw_/AROM_tot_) and push-off deficit (AW_st_). Those values were used as threshold to define the inclusion in a given subgroup in the baseline evaluation: NL=normal-like (squares), FD=foot-drop (circles), FD&POD=foot-drop and push-off deficit (triangles). The subgroup of surgically operated patients is evidenced with a dotted ellipse.

At individual level, a change greater than the SEM value evaluated for each parameter in a test-retest study on CMT patients (0.08 for AROM_ratio_ and 0.03 J/kg for AW_st_; [10,22]) was considered significant. To classify if a patient moved from the original cluster assigned in the baseline evaluation to another, a threshold correspondent to the 5^th^ percentile on the distribution of the relevant parameter in the controls’ group (AROM_ratio_ = 0.553 for the foot-drop deficit and AW_st_ = 0.218 J/kg for the push-off deficit) was adopted (see Figure 1).

## Results

Since the focus of the present paper is on the follow-up, we will not discuss here the comparison between controls and CMT1A patients in the baseline evaluation; however, significant differences, which were discussed in a previous paper [10], are reported in Table 1 as well.

**Table 1:** **Clinical scores and kinematic**/**kinetic parameters at the baseline and follow**-**up evaluation**

		**Controls**	**CMT1A**-**all**		**NL subgroup**		**FD subgroup**		**FD&POD subgroup**	
		**(N=18)**	**(N=16)**		**(N=7)**		**(N=6)**		**(N=3)**	
	**Session**		**Baseline**	**Follow-up**	**Baseline**	**Follow-up**	**Baseline**	**Follow-up**	**Baseline**	**Follow-up**
	Age [years]	11.0(3.3)	11.7(2.9)	13.2(2.9)	11.6(2.4)	13.0(2.4)	12.0(3.0)	13.6(2.9)	11.5(4.7)	12.9(4.8)
	Weight [kg]	41.4(14.3)	40.4(10.5)	45.9(11.3)	37.7(12.1)	46.1(15.7)	44.3(8.8)	47.4(6.3)	39.1(10.7)	42.8(10.1)
	Height [cm]	146(22)	148(15)	158(14)	145(16)	156(17)	152(12)	163(8)	147(22)	150(17)
*Clinical scores*										
	CMTES	-	3.4(2.3)	4.8(3.4)^#^	2.4(1.3)	3.5(2.1)	3.5(2.8)	5.2(4.3)	5.3(2.3)	7.0(4.3)
	ONLS	-	1.9(1.0)	2.4(1.0)	1.4(0.8)	2.4(1.0)	1.7(0.8)	2.0(1.1)	3.3(0.6)	3.3(0.6)
*Natural walking*										
	Gait speed [ms^-1^]	1.1(0.1)	1.2(0.1)	1.1(0.1)	1.1(0.1)	1.2(0.1)	1.2(0.1)	1.1(0.1)	1.0(0.1)	1.0(0.2)
	Gait speed/BH [%BH s^-1^]	77.0(7.0)	78.7(9.8)	74.7(13.3)	79.2(12.4)	77.7(13.9)	81.6(6.9)	72.6(9.1)^#^	71.8(6.2)	70.8(20.4)
	Cadence [steps min^-1^]	116(11)	117(10)	115(12)	115(13)	117(13)	119(7)	112(9)^#^	117(7)	117(19)
	Stride length [m]	1.2(0.2)	1.2(0.1)	1.2(0.1)	1.2(0.1)	1.3(0.1)^#^	1.2(0.1)	1.2(0.0)	1.1(0.1)	1.1(0.1)
	Stride length/BH [%BH]	79.8(4.1)	80.7(5.8)	78.3(7.9)^#^	82.0(5.0)	81.0(6.8)	82.1(5.3)	78.6(5.9)	75.1(6.7)	71.7(12.4)
	**AROM**_**ratio**_	0.65(0.09)	0.55(0.20) ^*^	0.51(0.21)	0.74(0.09)	0.63(0.18)	0.46(0.04)^a,b^	0.47(0.20)	0.26(0.03)^a,b,c^	0.27(0.09)
	**AW**_**st **_**[J/kg]**	0.36(0.10)	0.28(0.07) ^*^	0.28(0.06)	0.30(0.06)	0.29(0.05)	0.31(0.04)	0.30(0.04)	0.17(0.02)^a^	0.19(0.03)
*Toe*-*heel walking*										
	ATH_Δmean_	27.8(8.9)	16.9(7.6) ^*^	15.4(8.3)	20.7(7.6)	20.0(6.2)	17.7(5.9)^a^	15.7(7.8)	14.3(7.5)^a^	13.4(3.2)

The table shows clinical scores and kinematic/kinetic parameters at the baseline and follow-up evaluation for the whole group of CMT patients (CMT1A-all) and for the different subgroups (*NL* normal-like, *FD* Foot-drop, *FD*&*POD* Foot-drop and Push-off Deficit). Data from the control group are reported in the first column. Data are given in terms of mean and SD (in parentheses).

The two parameters used to classify patients into sub-groups based on the baseline evaluation are evidenced in bold (*AROM*_*ratio*_ foot-drop index, *AW*_*st*_ push-off deficit index). *BH* Body height, *Toe*-*heel* Toe- and Heel-walking trials. See the text (section Methods) for the meaning of biomechanical parameters.

^*^ Significant differences between controls and CMT1A-all group at baseline (p<0.05 T-test).

^a^ Significant differences between controls and CMT1A sub-groups at baseline (p<0.05 ANOVA and Tukey’s HSD post-hoc).

^b^ Significant differences between NL and FD or FD&POD group at baseline (p<0.05 ANOVA and Tukey’s HSD post-hoc).

^c^ Significant differences between FD and FD&POD group at baseline (p<0.05 ANOVA and Tukey’s HSD post-hoc).

^#^ Significant differences between baseline and follow-up evaluation (p<0.05 paired T-test for biomechanical indexes and Wilcoxon Signed Rank test for clinical scores).

Data from the 16 non-operated CMT1A participants showed that overall, a significant worsening of CMTES score (from 3.4±2.3 to 4.8±3.4, p<0.05) and a reduction of stride length of gait (80.9±5.9 to 78.4±8.1%BH, p<0.05) occurred between the baseline and the follow-up evaluation. No other significant difference between the two sessions could be identified. However, by inspecting the data of individual patients, different behaviors were observed as shown in Figure 1, which reports the individual changes of position in the plan of primary sign parameters (AROM_ratio_ and AW_st_). It resulted that all but one of the non-operated patients significantly changed at least one of the two parameters: 8 patients changed only one parameter and 7 both. Specifically, 11 participants significantly worsened at least one parameter (2 worsened both parameters while 5 improved the other parameter) and 9 significantly improved one parameter.

As regards clinical scores, 11 out of 16 participants did not change ONLS score and 4 did not change CMTES score. A change of 1 (minimum detectable change at individual level) in ONLS and CMTES score was found in 3 and 6 participants, respectively. No correlation (Rho absolute value always smaller than 0.5, p-value always greater than 0.09) between changes in biomechanical and clinical indexes was identified.

At sub-group level (see Table 1), patients originally belonging to the NL subgroup showed a trend towards a worsening of foot-drop deficit (−15%, from 0.74 to 0.63, p=0.12). Specifically, two patients (indicated with G and Q in Figure 1) showed at follow-up a foot-drop which moved them to the FD subgroup while the other five showed changes in primary parameters which were not enough to exit them from the normal-like subgroup. FD patients, as a group, significantly decreased gait velocity and cadence but did not change primary signs, although two opposite behaviors could be identified: three participants increased foot-drop index (patients B, I, N) allowing them to be re-classified into the NL subgroup, the other three worsened foot-drop and/or push-off deficit (A, K, O) although not moving to the worst FD&POD subgroup. Finally, non-operated FD&POD patients (D, F, H) did not change their abnormal condition as a sub-group (see Table 1). No significant changes in clinical scores were showed by any sub-group.

**Figure 2:**
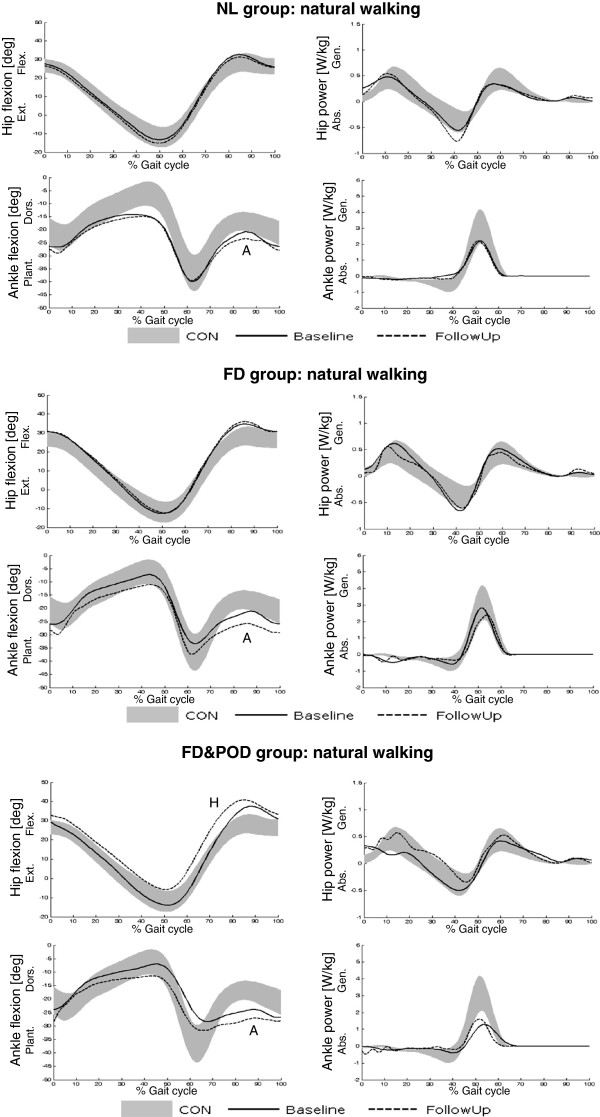
**Pattern of sagittal angular displacement (left panel) and power (right panel) of hip and ankle joints during natural walking for each CMT1A subgroup based on clusterization in the baseline evaluation: comparison between inter-subject average profiles at baseline (solid line) and follow-up (dotted line) evaluations.** Letters ‘A’ and ‘H’ indicate the main changes (see text). Data of the healthy group are reported as control reference (grey area, mean ±1SD). NL: normal-like, FD: foot-drop, FD&POD: foot-drop and push-off deficit.

In Figure 2 the time course of ankle and hip sagittal angle and power during natural walking trials are reported for all non-operated CMT1A patients, grouped according to the baseline evaluation. For each subgroup the comparison between inter-subject average profiles at baseline and follow-up evaluations are shown, together with the control range (grey area, mean ±1SD).

**Figure 3:**
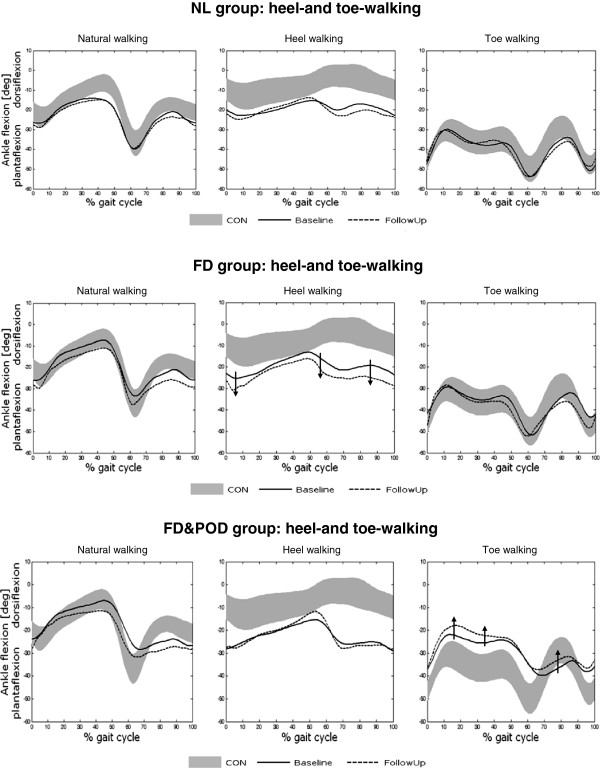
**Patterns of ankle flexion-extension angle during natural walking (left panel), heel-walking (center panel) and toe-walking (right panel) for each CMT1A subgroup: comparison between inter-subject average profiles at baseline (solid line) and follow-up (dotted line) evaluations.** Data of the healthy group is reported as control reference (grey area, mean ±1SD). Arrows show the main changes between baseline and follow-up evaluations in heel- and toe-walking.

The most significant changes in average joint kinematic/kinetic profiles between baseline and follow-up evaluations occurred at:

1) ankle joint, which slightly worsened foot-drop (indicated with ‘A’ in Figure 2) in all subgroups;

2) hip joint angle, which moved towards exaggerated flexion (H) in FD&POD patients;

As regards toe- and heel-walking trials, shown in Figure 3, all CMT1A subgroups showed abnormal ankle angle patterns at the baseline, including the NL subgroup who showed a normal pattern during natural walking but a less dorsiflexed ankle during heel-walking. In the follow-up session a worsening in heel-walking was shown by FD subgroup and in toe-walking by FD&POD subgroup (indicated by arrows in Figure 3). As a consequence, all sub-groups showed a trend towards a worsening of the ATH_Δmean_ in the follow-up evaluation with respect to baseline, although no one reached statistical significance (see Table 1, last row).

Due to the small number of patients who underwent foot surgery between the baseline and the follow-up evaluation, their results are here presented only descriptively. Specific surgical procedures performed on the three operated subjects were calcaneal osteotomy, extension osteotomy of the 1^st^ metatarsal head, and/or plantar fascia release.

All 3 patients improved the push-off index (see Figure 1), one of whom (patient M) improved also the foot-drop index and moved from the FD&POD to the normal-like subgroup. Conversely, ONLS clinical score did not change and CMTES worsened in all cases with a change of 1, 3 and 4 points respectively in patient M, E and L.

**Figure 4:**
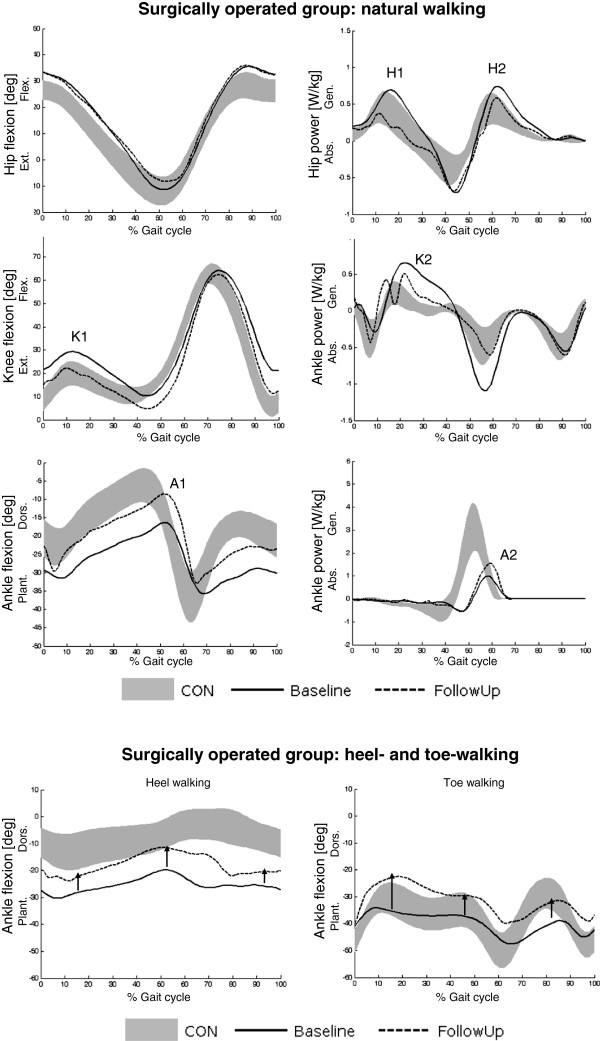
**Patterns of sagittal angular displacement (left panels) and power (right panels) of hip and ankle joints during natural walking for the subgroup of surgically operated CMT1A children: comparison between inter-subject average profiles at baseline (solid line) and follow-up (dotted line) evaluations.** Letters ‘A’, ‘H’ and ‘K’ indicate the main changes (see text). The bottom panels show, for the same subgroup of patients, the patterns of ankle flexion-extension angle during heel-walking (left panel) and toe-walking (right panel). Arrows show the shift of ankle angular pattern between baseline and follow-up evaluations. Data of the healthy group are reported as control reference (grey area, mean ±1SD).

Some changes in gait kinematics and kinetics were common to all 3 operated patients, as shown by inter-subject average profiles reported in Figure 4. Specifically they showed:

improvement of dorsiflexion during the whole gait cycle (A1) and increase of power production at push-off (A2);

improvement of knee joint kinematic and kinetic profiles in terms of knee yielding normalization (K1) and reduction of exaggerated power peak values at mid stance (K2) and initial swing (K3);

decrease of hip power exaggerated peak values at early/mid stance (H1) and initial swing (H2);

improvement of dorsiflexion in heel-walking and a reduction of plantarflexion in toe-walking.

## Discussion

Due to the very slowly rate of progression of the disease, monitoring the changes occurring in locomotor functions on patients with CMT1A is a challenging task. In a natural history study of 72 adult patients with CMT1A disease Shy et al. [12] reported an average CMTNS rate of worsening of 0.686 points per year. Subsequent studies found smaller values of 0.5 [23] and 0.3 [13] points per year. Finally, a recent 2 years clinical trial [24] found an average worsening of 0.23 points per year in the placebo group of 133 adult patients with CMT1A. Considering that the worst score of CMTNS is 36 points, it means that the average annual rate of change reported in literature ranges between 0.64% and 1.91% of maximal score. These small values are a concern for natural history studies and clinical trials in CMT1A disease, particularly in young patients where possible disease-related changes might be influenced by growing. Indeed, specific CMT pediatric score (CMTPedS) is being developed [7]. Results of the present study on children with CMT1A showed that after 18 months, besides a worsening of CMTES score, a significant reduction in stride length was identified through gait analysis in non-operated patients. Moreover, at individual level, all but one patients significantly changed at least one of the biomechanical parameters related to disease-specific distal deficits (i.e. foot-drop and push-off deficit) used to classify gait patterns, while clinical scores either did not change (ONLS) or only minimally worsened (CMTES) for most of the participants. This different degree of sensitivity between clinical and instrumental evaluation, may explain why we did not find any correlation between changes in biomechanical parameters and changes in clinical scores.

Although the group of non-operated CMT1A participants showed a slight reduction of stride length, indicating a general tendency of locomotor function to worsen, at individual level different behaviors were found: some patients clearly worsened their gait pattern in term of foot-drop and/or push-off deficit, other showed an improvement that, in three cases, even produced a reduction of foot-drop enough to move them from the FD subgroup to the normal-like subgroup. A considerable phenotypic variability within CMT1A is already known [1]: while some patients develop relevant weakness, require walking aids or even exceptionally become chair-bound, others are completely asymptomatic and unaware of being affected. Our results seem to indicate that, in young patients, a considerable inter-subject variability does not only characterize the degree of severity, but also its rate of progression, which we found quite different in our cohort of patients, with some patients even showing a walking deficit reduction in a 18 months time period. This result is supported by recent longitudinal data (derived from [7]) on 11 young patients with CMT1A, 5 of whom showed, after 1-year period, a decrease of disability as measured by CMTEPedS. Researches on genetic and/or environmental factors which influence disease severity are already in progress [1] and, to this aim, the availability of objective and sensitive indexes of disease-related deficit, like those presented in the present paper, is a crucial factor.

Although the number of operated patients is too small to conclude about efficacy of surgery, it is worth to mention that all of them showed a significant improvement in the push-off AW_st_ parameter. Since these patients originally belonged to the most severe group (FD&POD) and showed the worst push-off deficit within the entire group, this result seems to indicate a positive effect of surgery on push-off, crucial for walking efficiency. A possible explanation of this effect relies on the surgical correction of foot deformities at calcaneal, plantar and/or metatarsal level, which allows for a more physiological trajectory of the center of pressure and a lengthening of the level arm of ground reaction force with respect to the ankle at terminal stance. Indeed, at follow-up this group showed also a proximal improvement, secondary to distal surgery, in kinematic and kinetic profiles of knee and hip joints during natural walking. Finally, the release of plantar fascia might explain the reduced plantarflexion observed during toe-walking, where a stiff mid-foot is required.

Apart from surgery, no other factors (i.e. age, time since symptom onset, disease severity at the baseline, growth, participation in rehabilitation program or in sport activity) were identified to explain improvements in locomotor functions. However, we cannot be conclusive on the last mentioned factor, since the cohort of patients was coming from the whole Italian territory, therefore their therapeutic regimen and/or physical activity program was not under direct control and information were collected by a simple questionnaire. Moreover, standardized protocols for rehabilitation in CMT patients are still lacking, particularly for paediatric age. Further studies focused on those factors are needed to elucidate their effects on motor symptoms.

We found a rate of worsening of CMTES and ONLS of, respectively, 0.93 and 0.33 points per year, corresponding to 3.3% and 2.7% of maximal score, thus greater than those reported in literature for adult patients with CMT1A [12,13,23,24]. This might be due to the younger age of our sample or to other uncontrolled bias in patient selection, considering the relatively small size of our series, therefore results of the present study cannot be generalized to the entire CMT1A population. Another limitation of the present study, linked to the small sample size enrolled, is the underpowered sub-group statistical analysis that may have hidden differences between baseline and follow-up or among sub-groups.

Finally, it is to be underlined that some of the changes in gait patterns observed in young CMT patients in 18 months, might not be considered only as a direct consequence of disease progression, since the development of compensatory strategies and/or other factors associated to a possible atypical maturation of gait in presence of motor deficit cannot be excluded. In fact, while foot-drop worsening is reasonably the effect of disease-related weakening of dorsi-flexor muscles, push-off deficit may be attributed, in addition to weakening of plantar-flexors, also to a compensatory strategy aimed to find a trade-off between propulsive action, balance requirements and margin of safety during locomotion. Likewise, the trend towards exaggerated hip flexion during swing, showed by FD&POD patients at follow-up, can be explained as the development of a compensation to allow for foot clearance despite the worsening of foot-drop deficit. In this view, the specific analysis provided by instrumented gait analysis at different segments and joints is a valid tool for a comprehensive evaluation of locomotor function.

The large inter-subject variability on disease severity and on its rate of progression found in our young CMT patients, together with the possibility to distinguish primary signs from compensatory strategies, strengthen the use of gait analysis as a support for clinical decision making in the management of motor deficits in these patients: the knowledge of joint-specific locomotor abnormalities is expected to help in tailoring rehabilitation exercises, planning ankle-foot surgery and/or customizing lower limb orthosis for each individual.

## Conclusions

Our results showed that:

1) in young CMT1A patients subtle changes in gait parameters and kinematics/kinetics profiles, occurring in a 18 months period, can be identified instrumentally;

2) such changes show a large inter-subject variability, with some patients even improving their walking pattern, and are not correlated with changes in clinical scores; this result strengthens the use of gait analysis as a support for clinical decision making in the management of motor deficits in these patients;

3) there are anecdotal indications that corrective surgery of foot deformities may improve the push-off phase of gait, with secondary positive effects also at proximal joints.

Future studies with larger sample of patients and longer follow-up might verify if outcome measures provided by instrumented gait analysis are more sensitive and reliable than clinical scores for natural history studies and randomized clinical trials.

### Endnotes

^a^CMTES (CMT Examination Score) is the clinical component of the CMT Neuropathy Score (CMTNS), a validated composite scale for CMT [4], and has a score ranging from 0 (normal) to 28 (worst).

^b^ONLS (Overall Neuropathy Limitations Scale) is a scale to evaluate activity limitations in peripheral neuropathy [25] and has a score ranging from 0 (normal) to 12 (worst).

## Abbreviations

CMT: Charcot Marie tooth; CMT1A: Charcot Marie tooth type 1A; CMTES: Charcot Marie tooth examination score; CMTNS: Charcot Marie tooth neuropathy score; ONLS: Overall neuropathy limitation scale; NL: Normal-like; FD: Foot-drop; FD&POD: Foot-drop and push-off deficit; ROM: Range of motion; AROMratio: Ankle ROM ratio, index related to foot-drop deficit; AWst: Ankle positive work, index related to push-off deficit; SEM: Standard error of measurement

## Competing interests

The authors declared that they have no competing interest.

## Authors’ contributions

MF participated in study design, data elaboration and analysis of results; he performed study coordination and drafted the manuscript. TL participated in biomechanical data acquisition and elaboration, participated in the analysis of results, and performed statistical analysis. MR participated in study design, biomechanical data elaboration and in the analysis of results. IM participated in study design, patient selection, clinical evaluation and analysis of results. EP participated in study design, patient selection, clinical evaluation and analysis of results. DP participated in study design, led the clinical aspects of the study and participated in the analysis of results. All authors contributed to the revision of the draft, and have read and approved the final manuscript.
